# *catena*-Poly[[di-μ-aqua-bis[aqua­cobalt(II)]]-bis(μ_3_-1*H*-benzimidazole-5,6-dicarboxylato]

**DOI:** 10.1107/S1600536809005194

**Published:** 2009-02-21

**Authors:** Kai Xu, Li-Ping Yu

**Affiliations:** a Jiangxi University of Science and Technology, Ganzhou 341000, People’s Republic of China; b Department of Management and Engineering, Jiangxi V & T College of Communication, Nanchang 330013, People’s Republic of China

## Abstract

The title compound, [Co_2_(C_9_H_4_N_2_O_4_)_2_(H_2_O)_4_]_*n*_, is a one-dimensional polymeric complex with bridging 1*H*-benzimidazole-5,6-dicarboxyl­ate and aqua ligands. The Co^II^ cation has an octa­hedral coordination environment provided by an NO_5_ donor set. Adjacent polymeric chains extended along the [100] direction are linked by O—H⋯O and N—H⋯O hydrogen bonds, generating a three-dimensional network.

## Related literature

A dinuclear Co^II^ complex with a 1*H*-benzimidazole-5,6-dicarboxyl­ate anion as a bridging ligand was reported by Lo *et al.* (2007 ▶). For general information on polymeric coordination compounds, see: Barnett & Champness (2003 ▶); Eddaoudi *et al.* (2001 ▶); Kitagawa *et al.* (2004 ▶); Moulton & Zaworotko (2001 ▶); Roesky & Andruh (2003 ▶). 
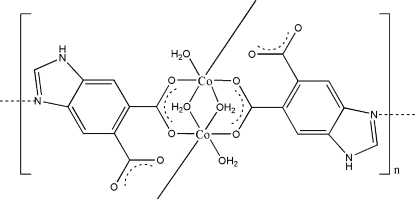


## Experimental

### 

#### Crystal data

[Co_2_(C_9_H_4_N_2_O_4_)_2_(H_2_O)_4_]
*M* *_r_* = 598.20
Monoclinic, 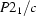

*a* = 8.8161 (8) Å
*b* = 9.1092 (6) Å
*c* = 13.0236 (13) Å
β = 97.693 (7)°
*V* = 1036.48 (16) Å^3^
*Z* = 2
Mo *K*α radiation
μ = 1.68 mm^−1^
*T* = 298 K
0.13 × 0.10 × 0.04 mm

#### Data collection

Bruker APEXII CCD diffractometer
Absorption correction: multi-scan (*SADABS*; Sheldrick, 1997 ▶) *T*_min_ = 0.801, *T*_max_ = 0.936
10641 measured reflections
2132 independent reflections
1843 reflections with *I* > 2σ(*I*)
*R*_int_ = 0.032

#### Refinement

*R*[*F* ^2^ > 2σ(*F* ^2^)] = 0.031
*wR*(*F* ^2^) = 0.084
*S* = 1.09
2132 reflections
178 parameters
7 restraints
H atoms treated by a mixture of independent and constrained refinement
Δρ_max_ = 0.24 e Å^−3^
Δρ_min_ = −0.25 e Å^−3^

### 

Data collection: *APEX2* (Bruker, 2004bbr id="bb12">); cell refinement: *SAINT-Plus* (Bruker, 2001 ▶); data reduction: *SAINT-Plus*; program(s) used to solve structure: *SHELXS97* (Sheldrick, 2008 ▶); program(s) used to refine structure: *SHELXL97* (Sheldrick, 2008 ▶); molecular graphics: *PLATON* (Spek, 2009 ▶); software used to prepare material for publication: *PLATON*.

## Supplementary Material

Crystal structure: contains datablocks I, global. DOI: 10.1107/S1600536809005194/gk2188sup1.cif

Structure factors: contains datablocks I. DOI: 10.1107/S1600536809005194/gk2188Isup2.hkl

Additional supplementary materials:  crystallographic information; 3D view; checkCIF report

## Figures and Tables

**Table 1:** Hydrogen-bond geometry (Å, °)

*D*—H⋯*A*	*D*—H	H⋯*A*	*D*⋯*A*	*D*—H⋯*A*
N2—H2⋯O3^i^	0.838 (17)	2.060 (18)	2.885 (3)	168 (3)
O5—H5*A*⋯O3	0.810 (16)	2.042 (18)	2.844 (2)	171 (3)
O5—H5*B*⋯O4^ii^	0.819 (16)	1.797 (17)	2.609 (2)	171 (3)
O6—H6*A*⋯O3^iii^	0.829 (17)	2.047 (19)	2.863 (2)	167 (3)
O6—H6*B*⋯O4^iv^	0.812 (17)	1.91 (2)	2.706 (3)	164 (3)

Symmetry codes: (i) 
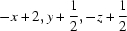
; (ii) 
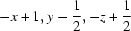
; (iii) 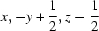; (iv) 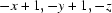.

**Fig. 1.**
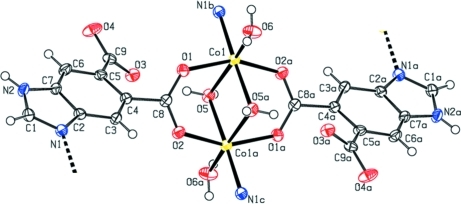
The molecular structure of the title compound showing the atom-numbering scheme. Displacement ellipsoids are drawn at the 50% probability level and H atoms are shown as small spheres of arbitrary radii. [Symmetry codes: (a) = 1 - x, -y, -z; (b) = x - 1, y, z and (c) = 2 - x, -y, -z.]

**Fig. 2.**
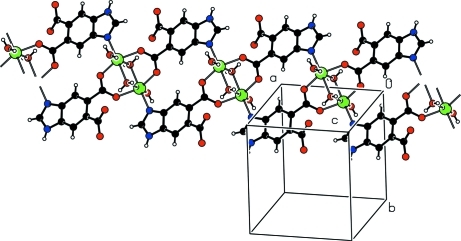
Part of the crystal structure of the title compound showing formation of the one-dimensional chain running parallel to the [100] direction..

**Fig. 3.**
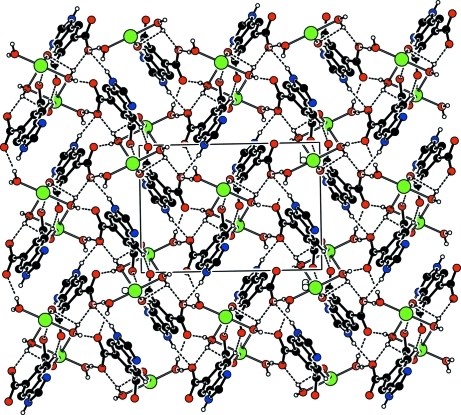
Part of the crystal structure showing the formation of the three-dimensional network. Hydrogen bonds are shown as dashed lines. Hydrogen atoms not involved in the motif have been omitted for clarity.

## supplementary crystallographic information

### Comment

Current interest in metal-organic coordination polymers is rapidly expanding owing to their intriguing structures and potential applications in the developments of optical, magnetic, superconductive and mineral materials (Moulton & Zaworotko, 2001; Eddaoudi *et al.*, 2001; Roesky & Andruh, 2003; Barnett & Champness, 2003; Kitagawa *et al.*, 2004). The assembly mode of coordination compounds is however strongly dependent on reaction conditions (pH, solvent, temperature, pressure, auxiliary ligands). Recently, Lo *et al.* (2007) reported the crystal structure of a dinuclear Co(II) compound obtained in the reaction of Co(NO_3_)_2_.6H_2_O and the multidentate ligand, 1*H*-benzimidazole-4,5-dicarboxylic acid (H_3_BIDC) in aqueous solution. However, when we applied hydrothermal method using the same substrates for the synthesis, a new compound, the title metal-organic polymer, was obtained (see Scheme).

The asymmetric unit of the title compound consists of one cobalt(II) cation, one HBIDC^2-^ ligand and two coordinating water molecules. The Co^II^ cation has an octahedral coordination environment which consists of one N atom, two O atoms from two different bidentate-bridging carboxylate groups, two O atoms from bridging water molecules and one O atom from a monodentate water molecule. The Co—O bond lengths range from 2.040 (1) to 2.250 (1) Å. The Co atoms are bridged into pairs by two carboxylate groups and two water molecules with the Co—Co distance of 3.126 (1) Å (Fig.1). These dimers are further connected by Co–N bonds to the benzimidazole units of other dimers forming a one-dimensional chain parallel to the [100] direction (Fig.2). The ligand bridging modes and assembly mode in the title compound are very much different from those observed in the dinuclear complex reported by Lo *et al.* (2007). The polymeric chains are linked together by several hydrogen bonds (Fig.3) forming a three-dimensional network. Further analysis reveals that this network is strengthened by a weak π···π interaction between phenyl rings of HBIDC^2-^ with face-to-face distances of 4.05 (1) Å and centroid- to- centroid distance of 3.81 (1) Å.

### Experimental

1*H*-Benzimidazole-5,6-dicarboxylic acid (0.083 g, 0.40 mmol) and Co(NO_3_)_2_.6H_2_O (0.24 g, 0.80 mmol) were dissolved in water (40 ml). pH of the solution was adjusted to 8 with 2*M *NaOH solution. The reaction mixture was placed in a Teflon reactor (15 mL) and was heated at 160 °C for 4 days, and then it was gradually cooled to room temperature at a rate of 10°C per hour. Purple crystals of (I) were obtained at the bottom of the reactor. Yield: 38% based on Co(NO_3_)_2_.6H_2_O.

### Refinement

All the H atoms bonded to C atoms were positioned geometrically with C–H = 0.93Å (aromatic) and refined in a riding mode with *U*_iso_(H) = 1.2*U*_eq_(C). H atoms bonded to O and N atoms were found in difference maps and the N—H and O—H distances were refined with restraints: N—H = 0.86 (2) Å, O—H = 0.82 (2) Å and H···H = 1.35 (2)° A for water molecules; their U values were set k times of the U_eq_ value of the carrier atom (k=1.2 for N and 1.5 for O bound H atoms).

### Crystal data

**Table:**

[Co_2_(C_9_H_4_N_2_O_4_)_2_(H_2_O)_4_]	*F*(000) = 604
*M**_r_* = 598.20	*D*_x_ = 1.917 Mg m^−^^3^
Monoclinic, *P*2_1_/*c*	Mo *K*α radiation, λ = 0.71073 Å
Hall symbol: -P 2ybc	Cell parameters from 3722 reflections
*a* = 8.8161 (8) Å	θ = 2.8–26.9°
*b* = 9.1092 (6) Å	µ = 1.68 mm^−^^1^
*c* = 13.0236 (13) Å	*T* = 298 K
β = 97.693 (7)°	Plate, pink
*V* = 1036.48 (16) Å^3^	0.13 × 0.10 × 0.04 mm
*Z* = 2	

### Data collection

**Table:**

Bruker APEX II CCD diffractometer	2132 independent reflections
Radiation source: fine-focus sealed tube	1843 reflections with *I* > 2σ(*I*)
graphite	*R*_int_ = 0.032
ω scan	θ_max_ = 26.5°, θ_min_ = 2.3°
Absorption correction: multi-scan (*SADABS*; Sheldrick, 1997)	*h* = −11→11
*T*_min_ = 0.801, *T*_max_ = 0.936	*k* = −11→10
10641 measured reflections	*l* = −16→16

### Refinement

**Table:**

Refinement on *F*^2^	Primary atom site location: structure-invariant direct methods
Least-squares matrix: full	Secondary atom site location: difference Fourier map
*R*[*F*^2^ > 2σ(*F*^2^)] = 0.031	Hydrogen site location: inferred from neighbouring sites
*wR*(*F*^2^) = 0.084	H atoms treated by a mixture of independent and constrained refinement
*S* = 1.09	*w* = 1/[σ^2^(*F*_o_^2^) + (0.0484*P*)^2^ + 0.4096*P*] where *P* = (*F*_o_^2^ + 2*F*_c_^2^)/3
2132 reflections	(Δ/σ)_max_ = 0.001
178 parameters	Δρ_max_ = 0.24 e Å^−^^3^
7 restraints	Δρ_min_ = −0.25 e Å^−^^3^

### Special details

**Table:**

Experimental. Crystal grew over two weeks.
Geometry. All e.s.d.'s (except the e.s.d. in the dihedral angle between two l.s. planes) are estimated using the full covariance matrix. The cell e.s.d.'s are taken into account individually in the estimation of e.s.d.'s in distances, angles and torsion angles; correlations between e.s.d.'s in cell parameters are only used when they are defined by crystal symmetry. An approximate (isotropic) treatment of cell e.s.d.'s is used for estimating e.s.d.'s involving l.s. planes.
Refinement. Refinement of *F*^2^ against ALL reflections. The weighted *R*-factor *wR* and goodness of fit *S* are based on *F*^2^, conventional *R*-factors *R* are based on *F*, with *F* set to zero for negative *F*^2^. The threshold expression of *F*^2^ > σ(*F*^2^) is used only for calculating *R*-factors(gt) *etc*. and is not relevant to the choice of reflections for refinement. *R*-factors based on *F*^2^ are statistically about twice as large as those based on *F*, and *R*- factors based on ALL data will be even larger.

### Fractional atomic coordinates and isotropic or equivalent isotropic displacement parameters (Å^2^)

**Table:**

	*x*	*y*	*z*	*U*_iso_*/*U*_eq_	
Co1	0.40810 (3)	0.14143 (3)	−0.03697 (2)	0.01731 (13)	
O1	0.62863 (17)	0.22091 (18)	0.00054 (13)	0.0215 (4)	
O2	0.76041 (18)	0.01355 (18)	0.04928 (13)	0.0244 (4)	
O3	0.66320 (18)	0.25466 (18)	0.22762 (12)	0.0231 (4)	
O4	0.7113 (2)	0.49433 (19)	0.24802 (15)	0.0329 (5)	
O5	0.47578 (18)	0.03826 (19)	0.11583 (12)	0.0205 (4)	
H5A	0.537 (3)	0.098 (3)	0.143 (2)	0.031*	
H5B	0.414 (3)	0.017 (3)	0.1548 (19)	0.031*	
O6	0.3858 (2)	0.2363 (2)	−0.18070 (14)	0.0321 (4)	
H6A	0.461 (3)	0.228 (3)	−0.213 (2)	0.048*	
H6B	0.345 (3)	0.315 (3)	−0.193 (3)	0.048*	
N1	1.2913 (2)	0.3115 (2)	0.03501 (15)	0.0202 (4)	
N2	1.2660 (2)	0.5038 (2)	0.13927 (16)	0.0226 (4)	
H2	1.286 (3)	0.584 (2)	0.170 (2)	0.027*	
C1	1.3576 (2)	0.4279 (3)	0.08205 (18)	0.0216 (5)	
H1	1.4578	0.4553	0.0765	0.026*	
C2	1.1427 (2)	0.3117 (3)	0.06301 (18)	0.0183 (5)	
C3	1.0189 (2)	0.2181 (3)	0.03410 (18)	0.0194 (5)	
H3	1.0255	0.1415	−0.0122	0.023*	
C4	0.8847 (2)	0.2441 (3)	0.07752 (17)	0.0184 (5)	
C5	0.8741 (3)	0.3586 (2)	0.15138 (18)	0.0186 (5)	
C6	0.9952 (3)	0.4545 (3)	0.17708 (18)	0.0223 (5)	
H6	0.9894	0.5312	0.2235	0.027*	
C7	1.1273 (2)	0.4306 (3)	0.12990 (18)	0.0198 (5)	
C8	0.7465 (3)	0.1518 (3)	0.03986 (17)	0.0186 (5)	
C9	0.7389 (3)	0.3696 (3)	0.21238 (18)	0.0192 (5)	

### Atomic displacement parameters (Å^2^)

**Table:**

	*U*^11^	*U*^22^	*U*^33^	*U*^12^	*U*^13^	*U*^23^
Co1	0.01344 (18)	0.0164 (2)	0.02276 (19)	−0.00114 (11)	0.00492 (12)	−0.00042 (12)
O1	0.0138 (8)	0.0209 (9)	0.0299 (9)	−0.0027 (6)	0.0033 (6)	0.0015 (7)
O2	0.0150 (8)	0.0181 (9)	0.0407 (10)	−0.0024 (6)	0.0061 (7)	−0.0040 (7)
O3	0.0224 (8)	0.0204 (9)	0.0281 (9)	−0.0014 (7)	0.0093 (7)	0.0020 (7)
O4	0.0360 (10)	0.0199 (10)	0.0484 (11)	0.0032 (8)	0.0269 (9)	−0.0022 (8)
O5	0.0177 (8)	0.0215 (9)	0.0239 (9)	−0.0042 (7)	0.0089 (7)	−0.0012 (7)
O6	0.0352 (11)	0.0314 (11)	0.0313 (10)	0.0100 (8)	0.0098 (8)	0.0104 (8)
N1	0.0133 (9)	0.0200 (11)	0.0279 (10)	−0.0017 (8)	0.0052 (8)	−0.0010 (8)
N2	0.0182 (10)	0.0188 (11)	0.0311 (11)	−0.0050 (8)	0.0045 (8)	−0.0071 (9)
C1	0.0131 (11)	0.0223 (13)	0.0299 (12)	−0.0033 (9)	0.0044 (9)	0.0010 (10)
C2	0.0129 (10)	0.0191 (12)	0.0238 (11)	0.0008 (9)	0.0053 (8)	0.0003 (9)
C3	0.0152 (11)	0.0174 (12)	0.0262 (12)	−0.0014 (9)	0.0055 (9)	−0.0056 (9)
C4	0.0126 (10)	0.0172 (12)	0.0260 (11)	−0.0007 (9)	0.0046 (8)	−0.0003 (10)
C5	0.0154 (11)	0.0170 (12)	0.0244 (11)	0.0033 (8)	0.0062 (9)	0.0002 (9)
C6	0.0214 (12)	0.0169 (12)	0.0296 (12)	0.0001 (9)	0.0073 (9)	−0.0059 (10)
C7	0.0143 (11)	0.0159 (12)	0.0289 (12)	−0.0021 (9)	0.0023 (9)	−0.0020 (10)
C8	0.0153 (11)	0.0189 (13)	0.0233 (11)	−0.0013 (9)	0.0086 (9)	−0.0033 (9)
C9	0.0155 (11)	0.0209 (13)	0.0219 (11)	0.0038 (9)	0.0056 (9)	0.0028 (9)

### Geometric parameters (Å, °)

**Table:**

Co1—O1	2.0709 (15)	N1—C2	1.406 (3)
Co1—O2^i^	2.0401 (16)	N2—C1	1.359 (3)
Co1—O5	2.2094 (17)	N2—C7	1.385 (3)
Co1—O5^i^	2.2503 (16)	N2—H2	0.838 (17)
Co1—O6	2.0472 (18)	C1—H1	0.9300
Co1—N1^ii^	2.144 (2)	C2—C3	1.396 (3)
O1—C8	1.263 (3)	C2—C7	1.407 (3)
O2—C8	1.270 (3)	C3—C4	1.398 (3)
O3—C9	1.272 (3)	C3—H3	0.9300
O4—C9	1.263 (3)	C4—C5	1.430 (3)
O5—H5A	0.810 (16)	C4—C8	1.507 (3)
O5—H5B	0.819 (16)	C5—C6	1.386 (3)
O6—H6A	0.829 (17)	C5—C9	1.521 (3)
O6—H6B	0.812 (17)	C6—C7	1.404 (3)
N1—C1	1.321 (3)	C6—H6	0.9300
			
O2^i^—Co1—O6	103.92 (8)	C1—N2—H2	127.0 (19)
O2^i^—Co1—O1	155.68 (7)	C7—N2—H2	126.0 (19)
O6—Co1—O1	92.30 (7)	N1—C1—N2	113.83 (19)
O2^i^—Co1—N1^ii^	98.46 (7)	N1—C1—H1	123.1
O6—Co1—N1^ii^	95.85 (8)	N2—C1—H1	123.1
O1—Co1—N1^ii^	97.75 (7)	C3—C2—N1	130.6 (2)
O2^i^—Co1—O5	83.25 (7)	C3—C2—C7	119.97 (19)
O6—Co1—O5	169.87 (7)	N1—C2—C7	109.4 (2)
O1—Co1—O5	78.72 (6)	C2—C3—C4	117.5 (2)
N1^ii^—Co1—O5	90.07 (7)	C2—C3—H3	121.3
O2^i^—Co1—O5^i^	80.31 (6)	C4—C3—H3	121.3
O6—Co1—O5^i^	83.31 (7)	C3—C4—C5	122.0 (2)
O1—Co1—O5^i^	83.79 (6)	C3—C4—C8	117.7 (2)
N1^ii^—Co1—O5^i^	178.28 (6)	C5—C4—C8	120.16 (19)
O5—Co1—O5^i^	90.98 (6)	C6—C5—C4	120.3 (2)
C8—O1—Co1	128.08 (15)	C6—C5—C9	117.67 (19)
C8—O2—Co1^i^	128.23 (15)	C4—C5—C9	121.7 (2)
Co1—O5—Co1^i^	89.02 (6)	C5—C6—C7	117.0 (2)
Co1—O5—H5A	101 (2)	C5—C6—H6	121.5
Co1^i^—O5—H5A	112 (2)	C7—C6—H6	121.5
Co1—O5—H5B	123 (2)	N2—C7—C6	131.4 (2)
Co1^i^—O5—H5B	119.5 (19)	N2—C7—C2	105.54 (19)
H5A—O5—H5B	110 (2)	C6—C7—C2	123.0 (2)
Co1—O6—H6A	116 (2)	O1—C8—O2	126.7 (2)
Co1—O6—H6B	123 (2)	O1—C8—C4	116.1 (2)
H6A—O6—H6B	110 (2)	O2—C8—C4	117.3 (2)
C1—N1—C2	104.40 (19)	O4—C9—O3	123.5 (2)
C1—N1—Co1^iii^	125.01 (15)	O4—C9—C5	117.0 (2)
C2—N1—Co1^iii^	129.27 (16)	O3—C9—C5	119.4 (2)
C1—N2—C7	106.8 (2)		
			
O2^i^—Co1—O1—C8	0.0 (3)	C8—C4—C5—C9	14.2 (3)
O6—Co1—O1—C8	−132.30 (18)	C4—C5—C6—C7	−1.8 (3)
N1^ii^—Co1—O1—C8	131.48 (18)	C9—C5—C6—C7	171.8 (2)
O5—Co1—O1—C8	42.96 (18)	C1—N2—C7—C6	175.5 (3)
O5^i^—Co1—O1—C8	−49.29 (18)	C1—N2—C7—C2	−1.3 (3)
O2^i^—Co1—O5—Co1^i^	80.12 (6)	C5—C6—C7—N2	−179.3 (2)
O6—Co1—O5—Co1^i^	−55.5 (4)	C5—C6—C7—C2	−3.0 (4)
O1—Co1—O5—Co1^i^	−83.47 (6)	C3—C2—C7—N2	−177.5 (2)
N1^ii^—Co1—O5—Co1^i^	178.64 (6)	N1—C2—C7—N2	1.7 (3)
C2—N1—C1—N2	0.5 (3)	C3—C2—C7—C6	5.4 (4)
Co1^iii^—N1—C1—N2	−167.35 (16)	N1—C2—C7—C6	−175.5 (2)
C7—N2—C1—N1	0.5 (3)	Co1—O1—C8—O2	12.4 (3)
C1—N1—C2—C3	177.7 (3)	Co1—O1—C8—C4	−168.66 (14)
Co1^iii^—N1—C2—C3	−15.1 (4)	Co1^i^—O2—C8—O1	−15.5 (3)
C1—N1—C2—C7	−1.4 (3)	Co1^i^—O2—C8—C4	165.65 (14)
Co1^iii^—N1—C2—C7	165.80 (16)	C3—C4—C8—O1	−121.1 (2)
N1—C2—C3—C4	178.3 (2)	C5—C4—C8—O1	55.9 (3)
C7—C2—C3—C4	−2.7 (3)	C3—C4—C8—O2	57.9 (3)
C2—C3—C4—C5	−2.0 (3)	C5—C4—C8—O2	−125.1 (2)
C2—C3—C4—C8	174.9 (2)	C6—C5—C9—O4	29.5 (3)
C3—C4—C5—C6	4.4 (4)	C4—C5—C9—O4	−156.9 (2)
C8—C4—C5—C6	−172.5 (2)	C6—C5—C9—O3	−148.4 (2)
C3—C4—C5—C9	−169.0 (2)	C4—C5—C9—O3	25.1 (3)

Symmetry codes: (i) −*x*+1, −*y*, −*z*; (ii) *x*−1, *y*, *z*; (iii) *x*+1, *y*, *z*.

### Hydrogen-bond geometry (Å, °)

**Table:**

*D*—H···*A*	*D*—H	H···*A*	*D*···*A*	*D*—H···*A*
N2—H2···O3^iv^	0.84 (2)	2.06 (2)	2.885 (3)	168 (3)
O5—H5A···O3	0.81 (2)	2.04 (2)	2.844 (2)	171 (3)
O5—H5B···O4^v^	0.82 (2)	1.80 (2)	2.609 (2)	171 (3)
O6—H6A···O3^vi^	0.83 (2)	2.05 (2)	2.863 (2)	167 (3)
O6—H6B···O4^vii^	0.81 (2)	1.91 (2)	2.706 (3)	164 (3)
C3—H3···O2^viii^	0.93	2.46	3.160 (3)	133

Symmetry codes: (iv) −*x*+2, *y*+1/2, −*z*+1/2; (v) −*x*+1, *y*−1/2, −*z*+1/2; (vi) *x*, −*y*+1/2, *z*−1/2; (vii) −*x*+1, −*y*+1, −*z*; (viii) −*x*+2, −*y*, −*z*.
